# Supplementary material for: Evolutionary insights into the emergence of virulent Leptospira spirochetes
Source: PLoS Pathog. 2024 Jul 17;20(7):e1012161. doi: 10.1371/journal.ppat.1012161 (PMC11285912; doi:10.1371/journal.ppat.1012161)
Supplement: S2 Table — (DOC) [file ppat.1012161.s002.doc]

| **old locus tag *L. interrogans serovar Manilae* UP-MMC-NIID LP** | **old locus tag *L. interrogans serovar Copenhageni* Fiocruz L1-130** | **Reference** |
| --- | --- | --- |
| LIMLP_10145 | LIC12032 | ^1^ |
| LIMLP_15405 |  | ^2–4^ |
| LIMLP_15415 |  | ^2–4^ |
| LIMLP_03070 | LIC12875 | ^5^ |
| LIMLP_06640 | LIC11360 | ^6,7^ |
| LIMLP_05255 | LIC11087 | ^8^ |
| LIMLP_09705 | LIC11947 | ^9,10^ |
| LIMLP_16990 | LIC13322 | ^11^ |
| LIMLP_04780 | LIC12906 | ^11–13^ |
|  | LIC10997 | ^11^ |
| LIMLP_02010 | LIC13086 | ^14^ |
| LIMLP_08500 | LIC11711 | ^15,16^ |
| LIMLP_12950 | LIC12587 | ^16^ |
| LIMLP_16665 | LIC13259 | ^17^ |
| LIMLP_09740 | LIC11954 | ^18,19^ |
| LIMLP_15190 | LIC10508 | ^20,21^ |
| LIMLP_05865 | LIC10365 | ^22^ |
| LIMLP_15185 | LIC10507 | ^20,21^ |
| LIMLP_04025 | LIC12690 | ^23^ |
| LIMLP_06600 | LIC11352 | ^13,24^ |
| LIMLP_03665 | LIC12760 | ^25^ |
| LIMLP_17395 | LIC13411 | ^26^ |
| LIMLP_11665 | LIC12341 | ^26^ |
| LIMLP_07685 | LIC11574 | ^26^ |
| LIMLP_13280 | LIC10879 | ^27^ |
| LIMLP_07160 | LIC11469 | ^28^ |
| LIMLP_09115 | LIC11834 | ^29^ |
| LIMLP_11180 | LIC12253 | ^29^ |
| LIMLP_04845 | LIC11009 | ^7^ |
| LIMLP_14510 | LIC10645 | ^30^ |
| LIMLP_14055 | LIC10731 | ^30^ |
| LIMLP_11235 | LIC12263 | ^31^ |
| LIMLP_02125 | LIC13059 | ^27^ |
| LIMLP_09235 | LIC11859 | ^32^ |
| LIMLP_04660 | LIC10973 | ^33^ |
| LIMLP_15115 | LIC10524 | ^34^ |
| LIMLP_15240 | LIC10494 | ^13^ |
| LIMLP_03820 | LIC12730 | ^13^ |
| LIMLP_13750 | LIC10793 | ^13^ |
| LIMLP_00490 | LIC10091 | ^13^ |
| LIMLP_00285 | LIC10054 | ^13^ |
| LIMLP_11105 | LIC12238 | ^13^ |
| LIMLP_16490 | LIC10258 | ^35^ |
| LIMLP_03045 | LIC12880 | ^35^ |
| LIMLP_03370 | LIC12816 | ^34^ |
| LIMLP_13775 | LIC10788 | ^34^ |
| LIMLP_05790 | LIC11194 | ^34^ |
| LIMLP_03480 | LIC12795 | ^34^ |
| LIMLP_11985 | LIC12407 | ^34^ |
| LIMLP_04390 | LIC10920 | ^36^ |
| LIMLP_13605 | LIC10821 | ^37^ |
| LIMLP_17720 | LIC13479 | ^38^ |
| LIMLP_00265 | LIC10050 | ^38^ |
| LIMLP_18410 | LIC20111 | ^39^ |

1. Eshghi, A. *et al.* Leptospira interrogans Catalase Is Required for Resistance to H2O2 and for Virulence. *Infect. Immun.* **80**, 3892–3899 (2012).

2. Matsunaga, J. *et al.* Pathogenic Leptospira species express surface-exposed proteins belonging to the bacterial immunoglobulin superfamily. *Mol. Microbiol.* **49**, 929–945 (2003).

3. Castiblanco-Valencia, M. M. *et al.* Plasmin cleaves fibrinogen and the human complement proteins C3b and C5 in the presence of Leptospira interrogans proteins: A new role of LigA and LigB in invasion and complement immune evasion. *Immunobiology* **221**, 679–689 (2016).

4. Choy, H. A. *et al.* Physiological osmotic induction of Leptospira interrogans adhesion: LigA and LigB bind extracellular matrix proteins and fibrinogen. *Infect. Immun.* **75**, 2441–2450 (2007).

5. Wolff, D. G. *et al.* Interaction of Leptospira elongation factor Tu with plasminogen and complement factor H: a metabolic leptospiral protein with moonlighting activities. *PloS One* **8**, e81818 (2013).

6. Siqueira, G. H., Atzingen, M. V., de Souza, G. O., Vasconcellos, S. A. & Nascimento, A. L. T. O. Leptospira interrogans Lsa23 protein recruits plasminogen, factor H and C4BP from normal human serum and mediates C3b and C4b degradation. *Microbiol. Read. Engl.* **162**, 295–308 (2016).

7. Siqueira, G. H. *et al.* Characterization of three novel adhesins of Leptospira interrogans. *Am. J. Trop. Med. Hyg.* **89**, 1103–1116 (2013).

8. Souza, N. M. *et al.* Lsa30, a novel adhesin of Leptospira interrogans binds human plasminogen and the complement regulator C4bp. *Microb. Pathog.* **53**, 125–134 (2012).

9. Barbosa, A. S. *et al.* Functional characterization of LcpA, a surface-exposed protein of Leptospira spp. that binds the human complement regulator C4BP. *Infect. Immun.* **78**, 3207–3216 (2010).

10. da Silva, L. B. *et al.* Pathogenic Leptospira species acquire factor H and vitronectin via the surface protein LcpA. *Infect. Immun.* **83**, 888–897 (2015).

11. Fraga, T. R. *et al.* Immune evasion by pathogenic Leptospira strains: the secretion of proteases that directly cleave complement proteins. *J. Infect. Dis.* **209**, 876–886 (2014).

12. Verma, A. *et al.* LfhA, a Novel Factor H-Binding Protein of Leptospira interrogans. *Infect. Immun.* **74**, 2659–2666 (2006).

13. Vieira, M. L. *et al.* In Vitro Identification of Novel Plasminogen-Binding Receptors of the Pathogen Leptospira interrogans. *PLoS ONE* **5**, e11259 (2010).

14. Passalia, F. J., Heinemann, M. B., Vieira, M. L. & Nascimento, A. L. T. O. A Novel Leptospira interrogans Protein LIC13086 Inhibits Fibrin Clot Formation and Interacts With Host Components. *Front. Cell. Infect. Microbiol.* **11**, 708739 (2021).

15. Kochi, L. T., Fernandes, L. G. V. & Nascimento, A. L. T. O. Heterologous Expression of the Pathogen-Specific LIC11711 Gene in the Saprophyte L. biflexa Increases Bacterial Binding to Laminin and Plasminogen. *Pathogens* **9**, 599 (2020).

16. Kochi, L. T. *et al.* The interaction of two novel putative proteins of Leptospira interrogans with E-cadherin, plasminogen and complement components with potential role in bacterial infection. *Virulence* **10**, 734–753 (2019).

17. Cavenague, M. F. *et al.* Characterization of a novel protein of Leptospira interrogans exhibiting plasminogen, vitronectin and complement binding properties. *Int. J. Med. Microbiol. IJMM* **309**, 116–129 (2019).

18. Eshghi, A. *et al.* Pathogenic Leptospira interrogans Exoproteins Are Primarily Involved in Heterotrophic Processes. *Infect. Immun.* **83**, 3061–3073 (2015).

19. Nogueira, S. V. *et al.* Leptospira interrogans enolase is secreted extracellularly and interacts with plasminogen. *PloS One* **8**, e78150 (2013).

20. Siqueira, G. H. *et al.* The recombinant LIC10508 is a plasma fibronectin, plasminogen, fibrinogen and C4BP-binding protein of Leptospira interrogans. *Pathog. Dis.* **74**, ftv118 (2016).

21. Gómez, R. M. *et al.* Putative outer membrane proteins of Leptospira interrogans stimulate human umbilical vein endothelial cells (HUVECS) and express during infection. *Microb. Pathog.* **45**, 315–322 (2008).

22. Vieira, M. L. *et al.* A novel leptospiral protein increases ICAM-1 and E-selectin expression in human umbilical vein endothelial cells. *FEMS Microbiol. Lett.* **276**, 172–180 (2007).

23. Atzingen, M. V. *et al.* Lp95, a novel leptospiral protein that binds extracellular matrix components and activates e-selectin on endothelial cells. *J. Infect.* **59**, 264–276 (2009).

24. Sun, Z., Bao, L., Li, D., Huang, B. & Wu, B. Effect of Leptospira interrogans outer membrane proteins LipL32 on HUVEC. *Microb. Pathog.* **49**, 116–121 (2010).

25. Kassegne, K. *et al.* Identification of collagenase as a critical virulence factor for invasiveness and transmission of pathogenic Leptospira species. *J. Infect. Dis.* **209**, 1105–1115 (2014).

26. Evangelista, K. V. *et al.* Identification of cell-binding adhesins of Leptospira interrogans. *PLoS Negl. Trop. Dis.* **8**, e3215 (2014).

27. Pereira, P. R. M. *et al.* Multifunctional and Redundant Roles of Leptospira interrogans Proteins in Bacterial-Adhesion and fibrin clotting inhibition. *Int. J. Med. Microbiol. IJMM* **307**, 297–310 (2017).

28. Mendes, R. S. *et al.* The Novel Leptospiral Surface Adhesin Lsa20 Binds Laminin and Human Plasminogen and Is Probably Expressed during Infection▿. *Infect. Immun.* **79**, 4657–4667 (2011).

29. Domingos, R. F. *et al.* Features of two proteins of Leptospira interrogans with potential role in host-pathogen interactions. *BMC Microbiol.* **12**, 50 (2012).

30. Fernandes, L. G. V. *et al.* Functional and immunological evaluation of two novel proteins of Leptospira spp. *Microbiol. Read. Engl.* **160**, 149–164 (2014).

31. Pinne, M., Choy, H. A. & Haake, D. A. The OmpL37 surface-exposed protein is expressed by pathogenic Leptospira during infection and binds skin and vascular elastin. *PLoS Negl. Trop. Dis.* **4**, e815 (2010).

32. Cosate, M. R., Siqueira, G. H., de Souza, G. O., Vasconcellos, S. A. & Nascimento, A. L. T. O. Mammalian cell entry (Mce) protein of Leptospira interrogans binds extracellular matrix components, plasminogen and β2 integrin. *Microbiol. Immunol.* **60**, 586–598 (2016).

33. Fernandes, L. G. V. *et al.* OmpL1 Is an Extracellular Matrix- and Plasminogen-Interacting Protein of Leptospira spp. *Infect. Immun.* **80**, 3679–3692 (2012).

34. Vieira, M. L. *et al.* Plasminogen Binding Proteins and Plasmin Generation on the Surface of Leptospira spp.: The Contribution to the Bacteria-Host Interactions. *J. Biomed. Biotechnol.* **2012**, 758513 (2012).

35. Oliveira, R. *et al.* Characterization of novel OmpA-like protein of Leptospira interrogans that binds extracellular matrix molecules and plasminogen. *PloS One* **6**, e21962 (2011).

36. Rossini, A. D. *et al.* Identification of a novel protein in the genome sequences of Leptospira interrogans with the ability to interact with host’s components. *J. Microbiol. Immunol. Infect. Wei Mian Yu Gan Ran Za Zhi* **53**, 163–175 (2020).

37. Silva, L. P. *et al.* Evaluation of two novel leptospiral proteins for their interaction with human host components. *Pathog. Dis.* **74**, ftw040 (2016).

38. Teixeira, A. F. *et al.* Features of two new proteins with OmpA-like domains identified in the genome sequences of Leptospira interrogans. *PloS One* **10**, e0122762 (2015).

39. Eshghi, A. *et al.* A Putative Regulatory Genetic Locus Modulates Virulence in the Pathogen Leptospira interrogans. *Infect. Immun.* **82**, 2542–2552 (2014).
