# Supplementary material for: Evolutionary insights into the emergence of virulent Leptospira spirochetes
Source: PLoS Pathog. 2024 Jul 17;20(7):e1012161. doi: 10.1371/journal.ppat.1012161 (PMC11285912; doi:10.1371/journal.ppat.1012161)
Supplement: S7 Table — Data shown in the column “serum” correspond to deregulated genes of L. interrogans serovar Copenhageni incubated with 50% of normal guinea pig serum at 37°C for to 2 hr; heat-inactivated serum was used as a control. Data shown in the column “DMC” correspond to L. interrogans serovar Copenhageni grown in dialysis membrane chambers (DMC) into the peritoneal cavities of rats for 10 days; in vitro-cultivated leptospires at 30°C in EMJH supplemented with 1% rabbit serum was used as a control. Data shown in the column “physiological osmolarity” correspond to L. interrogans serovar Copenhageni cultivated in EMJH complemented with 120 nM NaCl for 20 hr at 30°C; L. interrogans cultivated in EMJH without 120 nM NaCl was used as control. The absence of significant deregulation of gene expression was annotated by “-“. (PDF) [file ppat.1012161.s007.pdf]

		Expression (Fold change)		
**ORF name**	**description**	**Serum**	**DMC**	**Physiological osmolarity**
LIMLP_03665	Collagenase (ColA)	-	49.03	-
LIMLP_03505	Gene family-encoded virulence-modifying proteins	-	-	-
LIMLP_11655	Gene family-encoded virulence-modifying proteins	2,81	-	5.96
LIMLP_10965	Putative lipoprotein	-	7.76	2.88
LIMLP_13525	Putative lipoprotein (leucin-rich repeat domain)	-	-	-
LIMLP_13605	Putative lipoprotein	-	-	-
LIMLP_09380	Uncharacterized protein	1,57	5.71	5.64
