# Supplementary figures and images for: Evolutionary insights into the emergence of virulent Leptospira spirochetes
Source: PLoS Pathog. 2024 Jul 17;20(7):e1012161. doi: 10.1371/journal.ppat.1012161 (PMC11285912; doi:10.1371/journal.ppat.1012161)

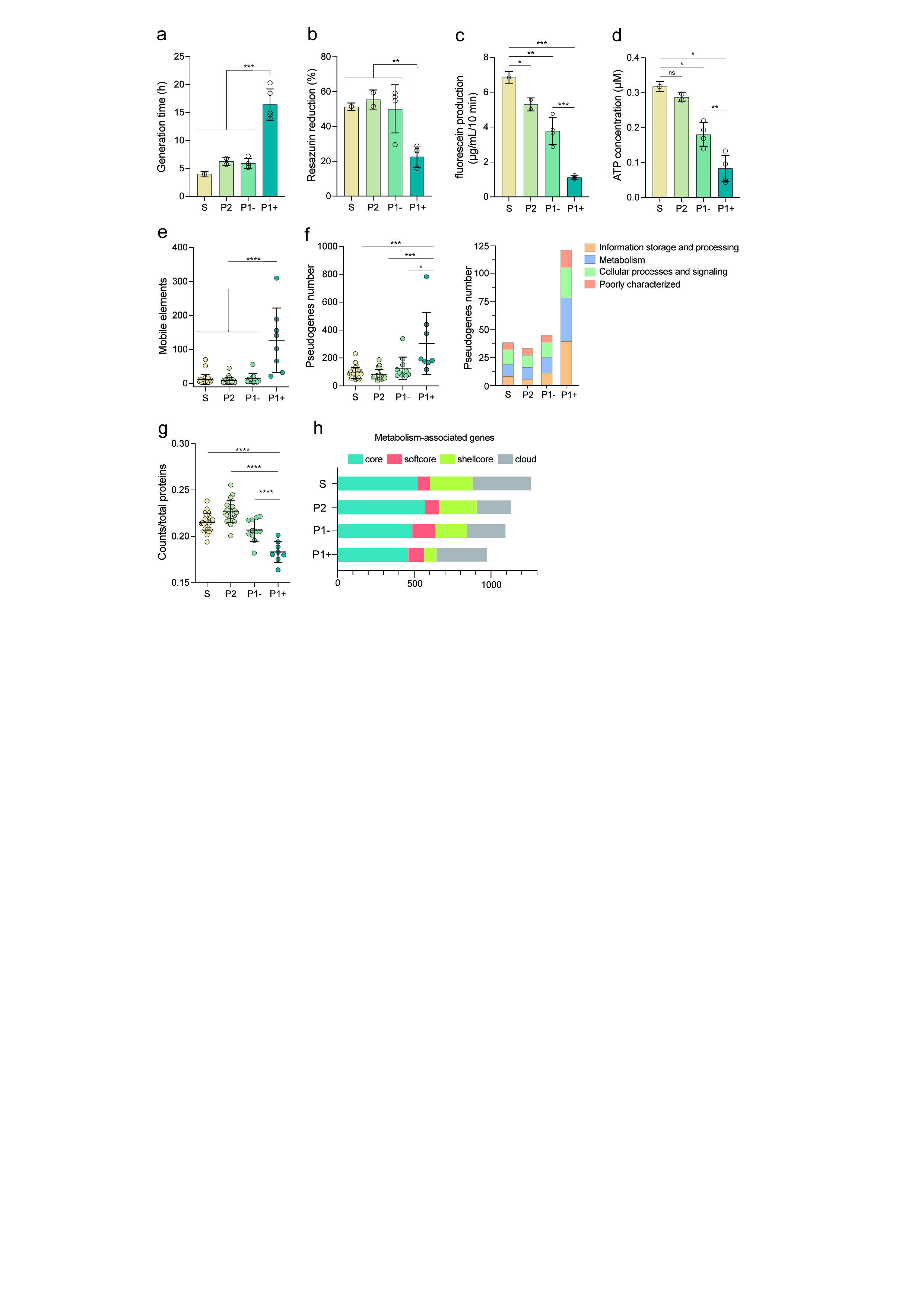

Supplement: S1 Fig — (a) Generation time of S, P2, P1- and P1+ species was calculated from growth curves in EMJH medium at 30°C. (b) Evaluation of the redox activity of Leptospira using the Alamar Blue assay. (c) Microbial activity of Leptospira was determined using the fluorescein diacetate assay. Bacteria were incubated with fluorescein diacetate during 10 min and then microbial activity was assessed through the ability of cells to hydrolyze fluorescein diacetate into fluorescein. (d) Determination of the ATP concentration of Leptospira spp. S: L. biflexa; P2: L. licerasiae, L. fluminis; P1- group: L. adleri, L. gomenensis, L. tipperyarensis, L. yasudae; P1+ group: L. interrogans, L. noguchii, L. weilii, L. santarosai, L. mayottensis. (e) Distribution of mobile elements in the genus Leptospira. (f) Distribution of pseudogenes in the genus Leptospira (left panel). Pseudogenes are distinguished using the COG classification (right panel). (g) Protein abundance distribution for the category Metabolism in the genus Leptospira. Each circle represents one Leptospira species. The abundance of proteins associated with the Metabolism category was calculated by the number of metabolic proteins divided by the total number of proteins for each Leptospira species. (h) Pan-genome distribution of metabolism-associated genes for groups S, P2, P1- and P1+ into four categories (core, softcore, shellcore and cloud). Unpaired two-tailed Student’s t test was used. *p< 0.01, **p<0.001, ***p<0.0001, ns: not significant. (TIF) [file ppat.1012161.s009.tif]

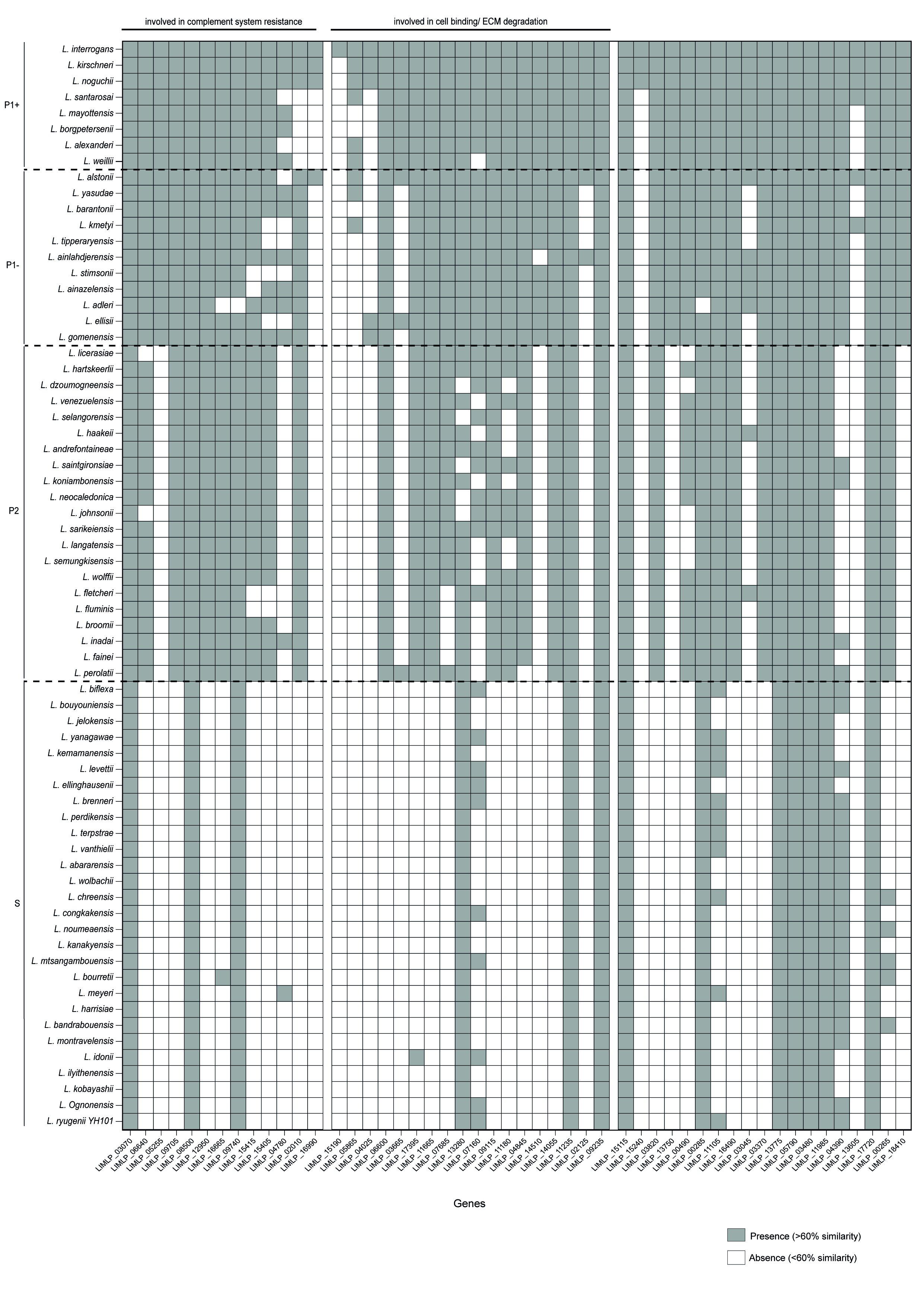

Supplement: S2 Fig — The presence/absence CDSs of all Leptospira species with percentage of similarity ⩾60% according to the protein sequence in L. interrogans. Grey and white squares denote the presence and absence of genes, respectively. (TIF) [file ppat.1012161.s010.tif]

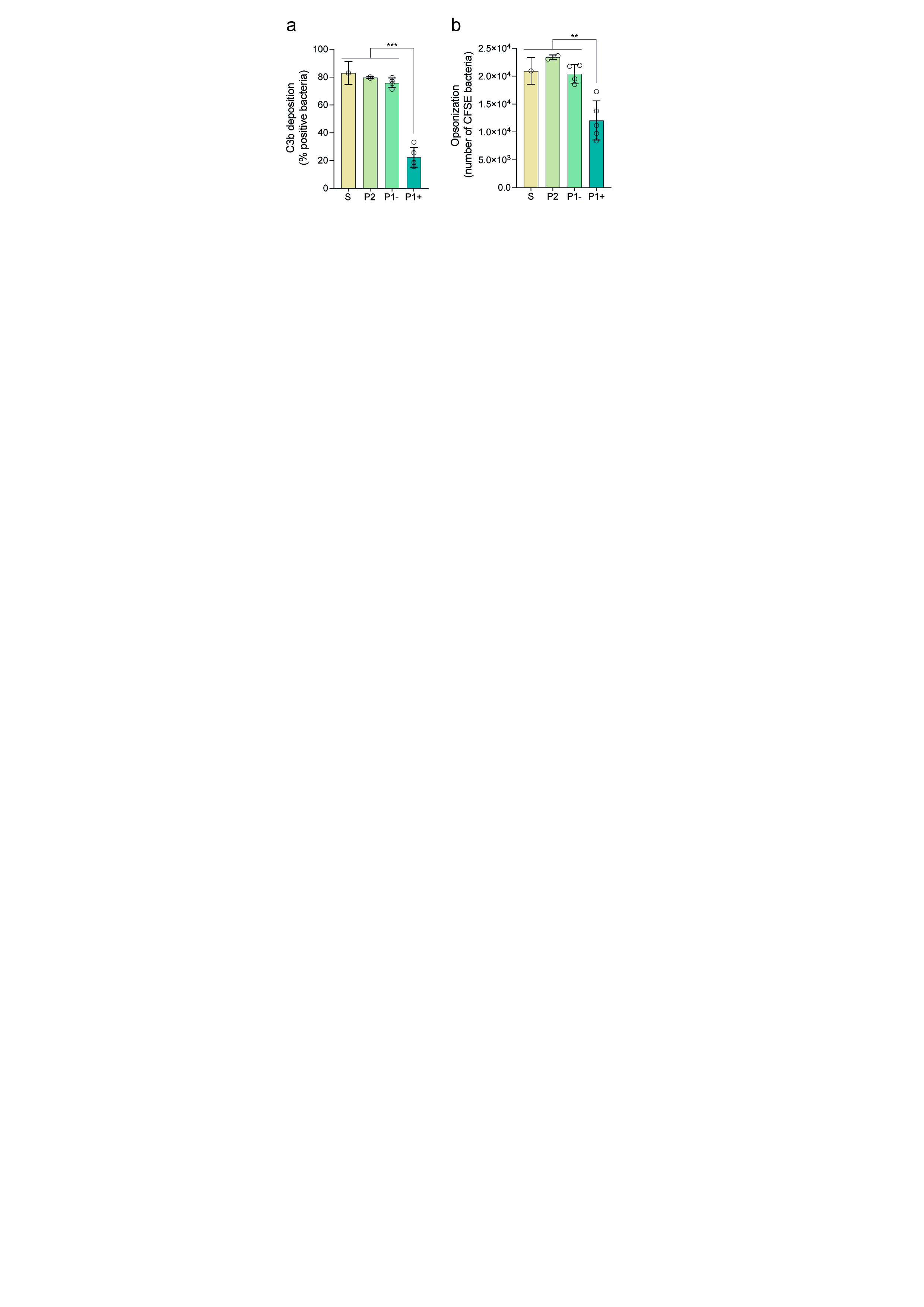

Supplement: S3 Fig — (a) Detection of C3b protein deposition in Leptospira by indirect immunofluorescence. Leptospira were stained with CFSE and then incubated with human serum for 30 min. Fixed cells were incubated with an anti-C3b antibody. Indirect immunofluorescence was quantified by flow cytometry. (b) Effect of human serum opsonization on macrophage uptake of Leptospira (MOI 1:100). CFSE-labelled Leptospira were incubated with human serum during 30 min. Bacteria were then added to macrophages (THP-1) during 2 hr following with 1 hr of gentamicin (100 μg/ml) treatment. Macrophages were lysed and intracellular bacteria were fixed. Number of CFSE bacteria was determined by flow cytometry. Opsonization was assessed by the ratio of human serum to inactivated-human serum. S: L. biflexa; P2: L. licerasiae, L. fluminis; P1- group: L. adleri, L. gomenensis, L. tipperyarensis, L. yasudae; P1+ group: L. interrogans, L. noguchii, L. weilii, L. santarosai, L. mayottensis. Unpaired two-tailed Student’s t test was used. **p<0.001, ***p<0.0001. (TIF) [file ppat.1012161.s011.tif]

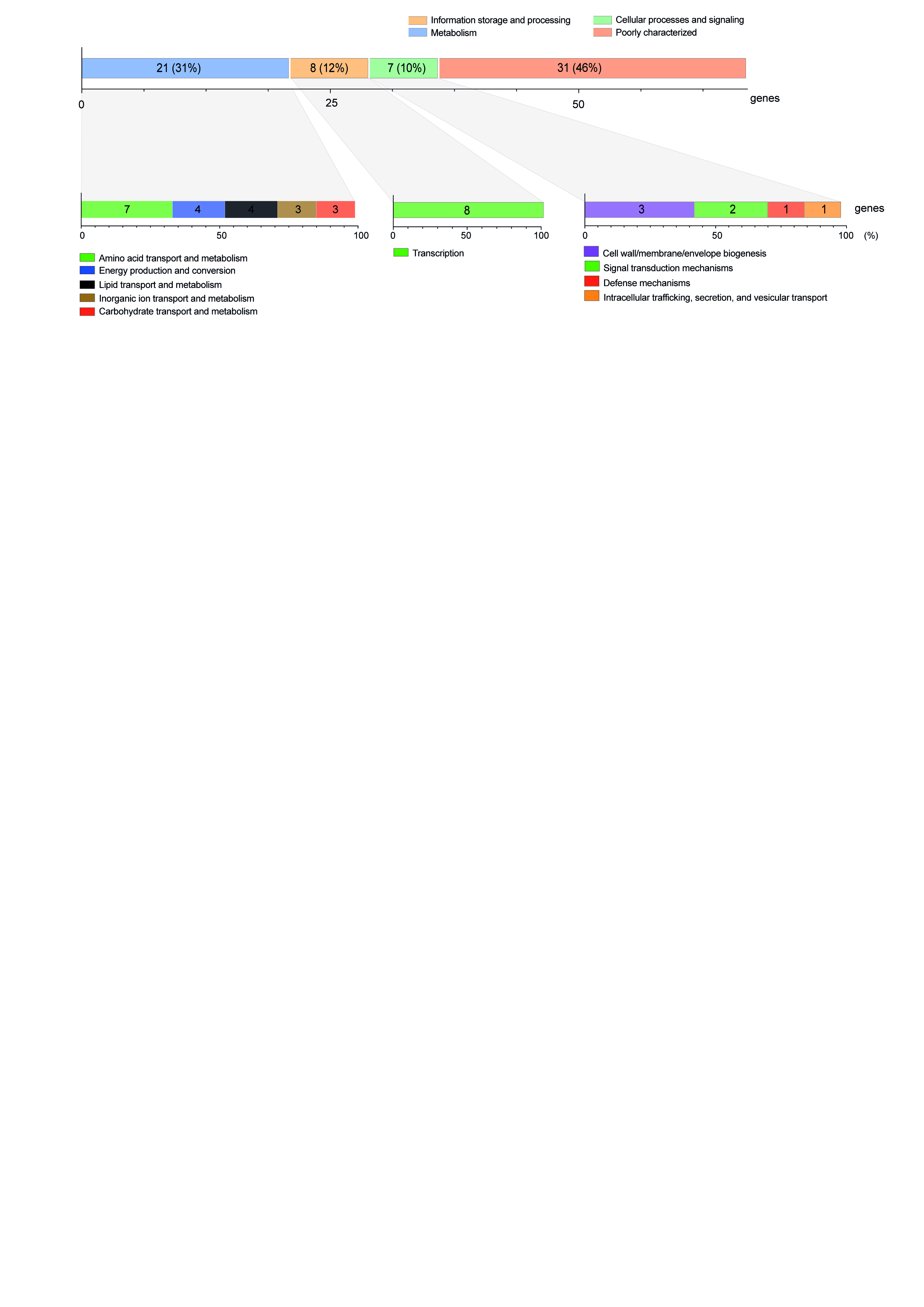

Supplement: S4 Fig — COG classification of genes lost in P1+ species. (TIF) [file ppat.1012161.s012.tif]

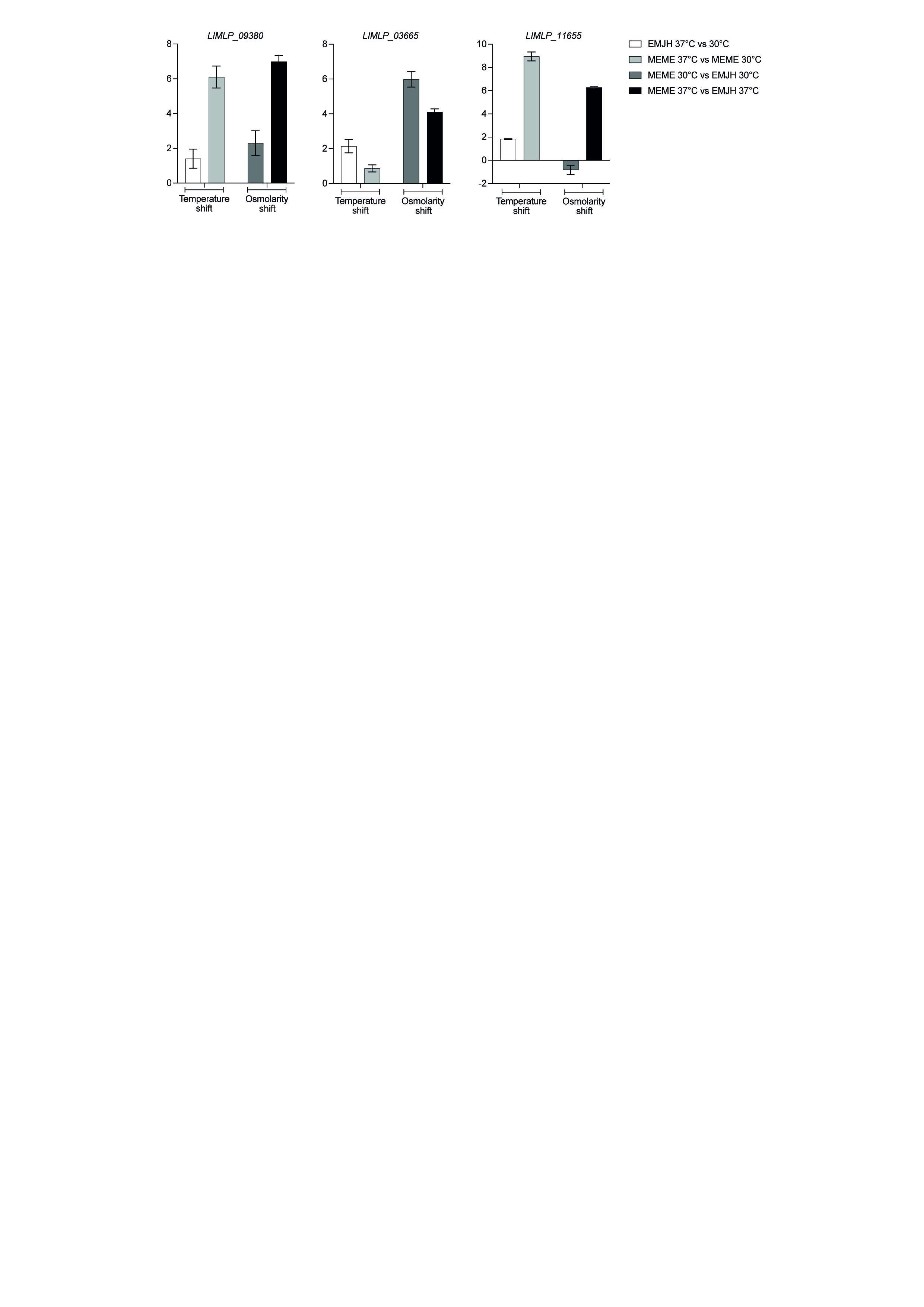

Supplement: S5 Fig — Relative expression of the genes in L. interrogans after overnight incubation in EMJH or MEME medium at 30°C or 37°C. Relative expression levels were normalized to the 16S RNA gene. (TIF) [file ppat.1012161.s013.tif]

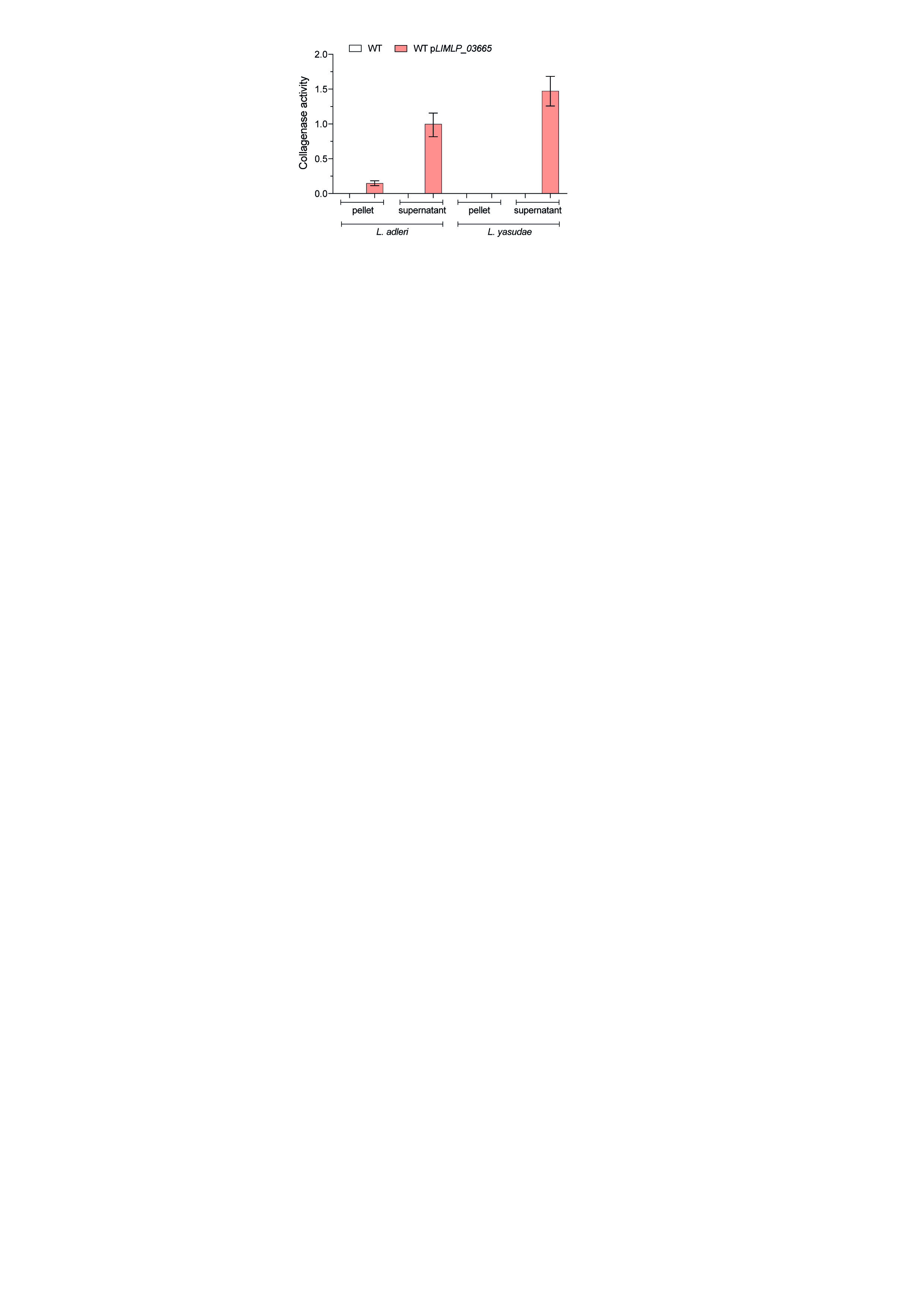

Supplement: S6 Fig — Measurement of collagenase activity in the supernatant or in total extracts of Leptospira. (TIF) [file ppat.1012161.s014.tif]

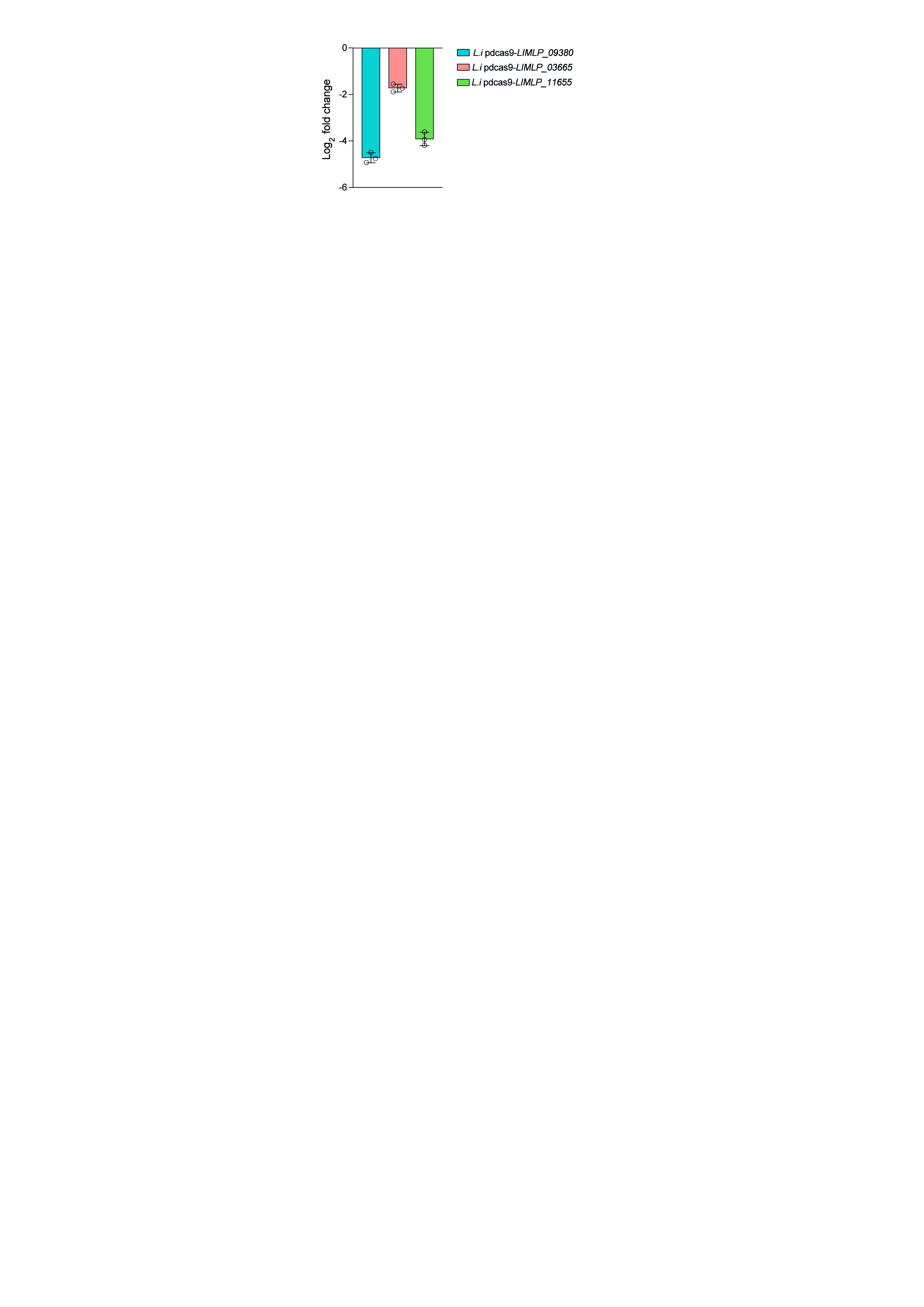

Supplement: S7 Fig — Total RNA was extracted from exponentially growing cultures of L. interrogans. Relative expression of genes was measured by RT-qPCR. Log2FC levels were normalized to the flaB2 gene and compared to L. interrogans WT. (TIF) [file ppat.1012161.s015.tif]

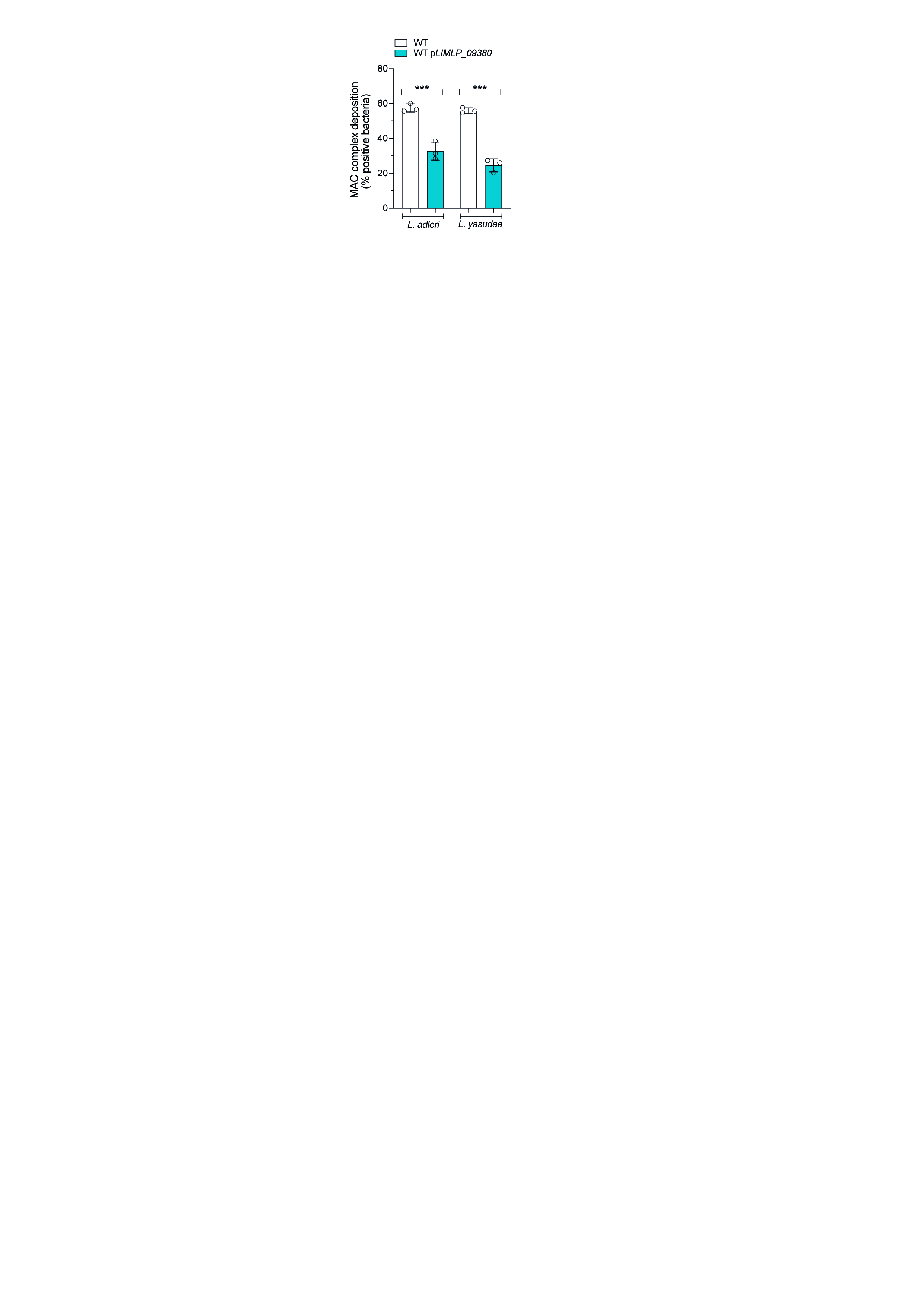

Supplement: S8 Fig — MAC deposition in P1- species (L. adleri and L. yasudae) expressing or not LIMLP_09380 was detected by indirect immunofluorescence. Leptospira were stained with CFSE and then incubated with human serum for 30 min. Fixed cells were incubated with an anti-MAC antibody (C5b9). Indirect immunofluorescence of MAC was quantified by flow cytometry. Unpaired two-tailed Student’s t test was used. *p< 0.01, **p<0.001, ***p<0.0001. Error bars represent the mean ± SD. (TIF) [file ppat.1012161.s016.tif]

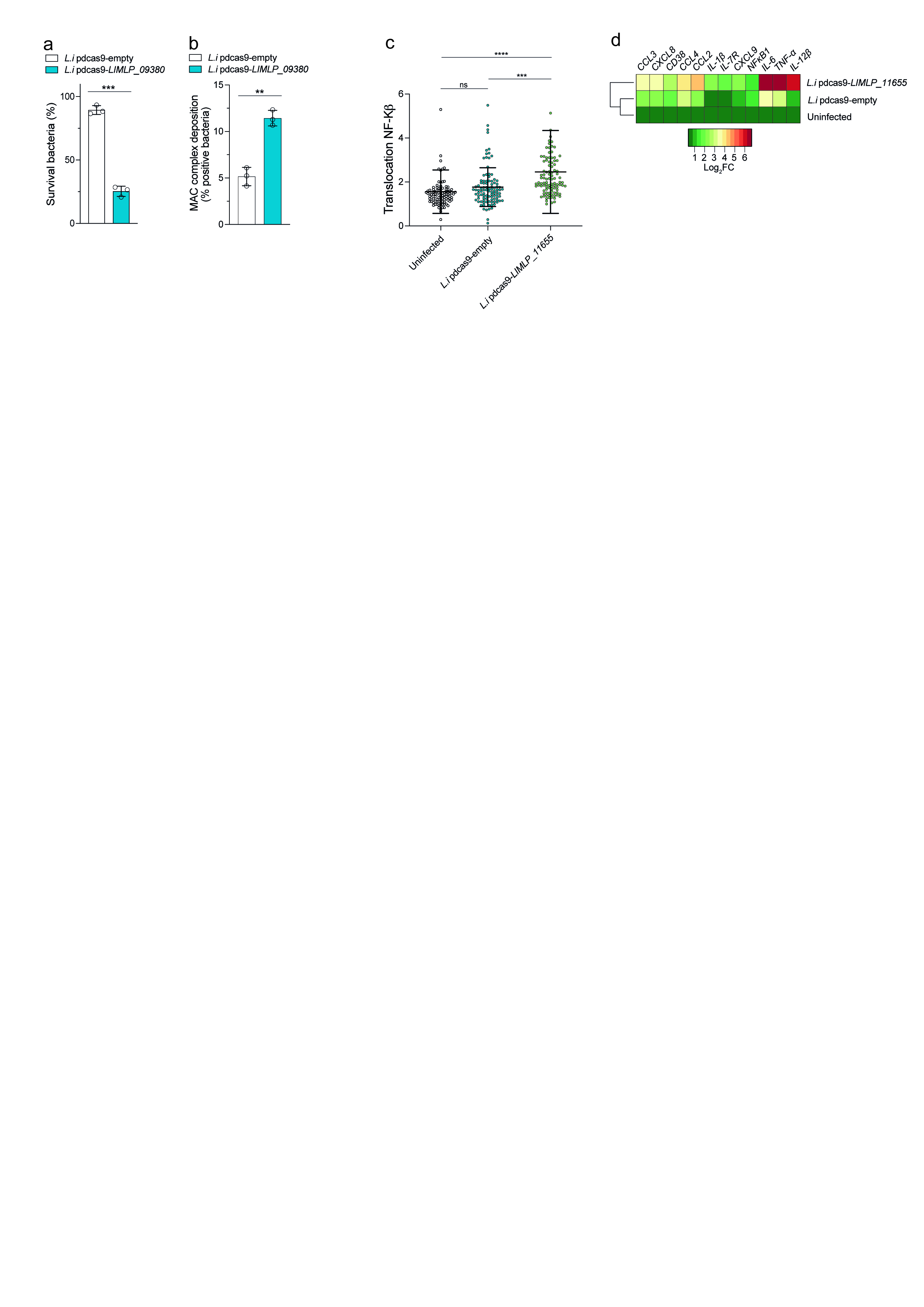

Supplement: S9 Fig — (a) The CRISPR-dcas9 knockdown of hyp/LIMLP_09380 in L. interrogans was incubated in 20% of normal or inactivated human serum for 2 hr. After incubation, the bacteria were enumerated by CFU and the percentage of surviving bacteria was normalized by Leptospira exposed to inactivated-human serum. Unpaired two-tailed Student’s t test was used. ***p<0.0001. Error bars represent the mean ± SD. (b) Detection by indirect immunofluorescence of MAC deposition in the CRISPR-dcas9 knockdown of hyp/LIMLP_09380 in L. interrogans. CFSE-labelled Leptospira were incubated with human serum for 30 min. Fixed cells were incubated with an anti-MAC antibody (C5b9). Indirect immunofluorescence of MAC was quantified by flow cytometry. Unpaired two-tailed Student’s t test was used. **p<0.001. Error bars represent the mean ± SD. (c) Ratio between nuclear and cytosolic NF-κB fluorescence intensity (n > 100 cells per condition, two-way ANOVA test; ****p<0,001; ns: not significant) in the CRISPR-dcas9 knockdown of VM/LIMLP_11655 in L. interrogans. (TIF) [file ppat.1012161.s017.tif]
